# Supplementary material for: DMRforPairs: identifying Differentially Methylated Regions between unique samples using array based methylation profiles
Source: BMC Bioinformatics. 2014 May 15;15:141. doi: 10.1186/1471-2105-15-141 (PMC4046028; doi:10.1186/1471-2105-15-141)
Supplement: Additional file 2 — DMRforPairs output for the comparison of A431-MCF7 and NA17018-NA17105. Please start from the HTML files in each folder. Available via the BMC Bioinformatics website. [file 1471-2105-15-141-S2.zip › 1394847754114233_MOESM2_ESM/A431_MCF7/figures/33617.pdf]

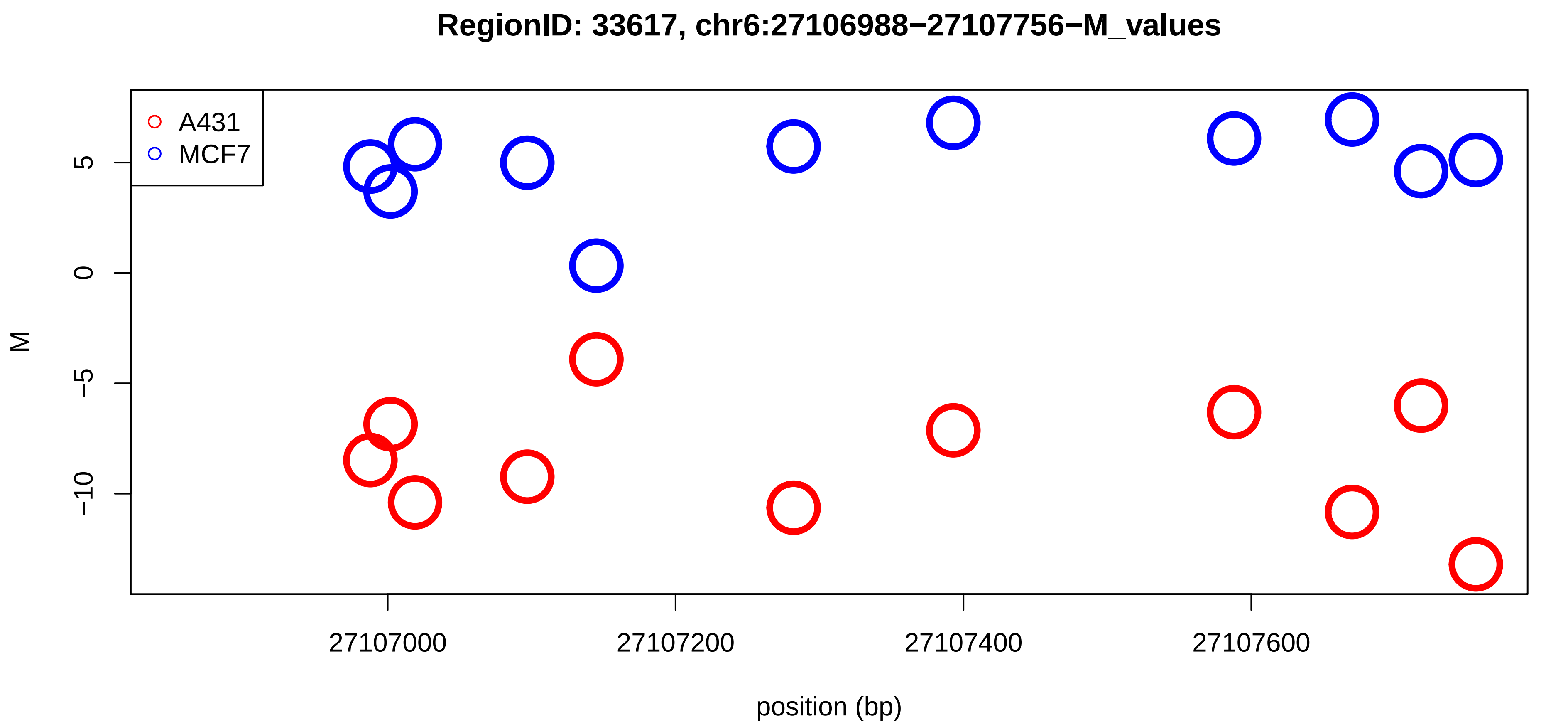

RegionID: 33617, chr6:27106988−27107756−M_values
A431
MCF7
M
5
0
−5
−10
27107000
27107200
27107400
27107600
position (bp)

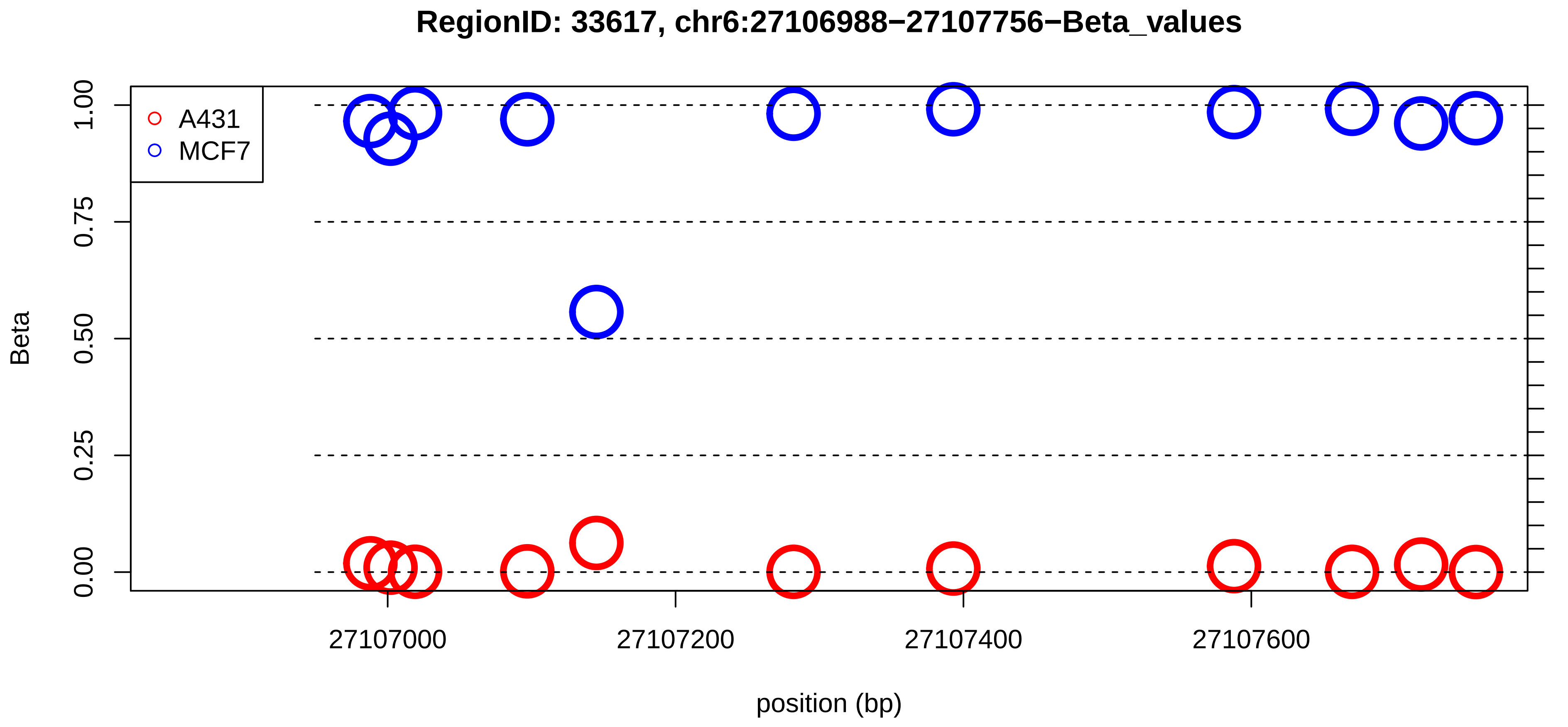

RegionID: 33617, chr6:27106988−27107756−Beta_values
A431
MCF7
Beta
1.00
0.75
0.50
0.25
0.00
27107000
27107200
27107400
27107600
position (bp)

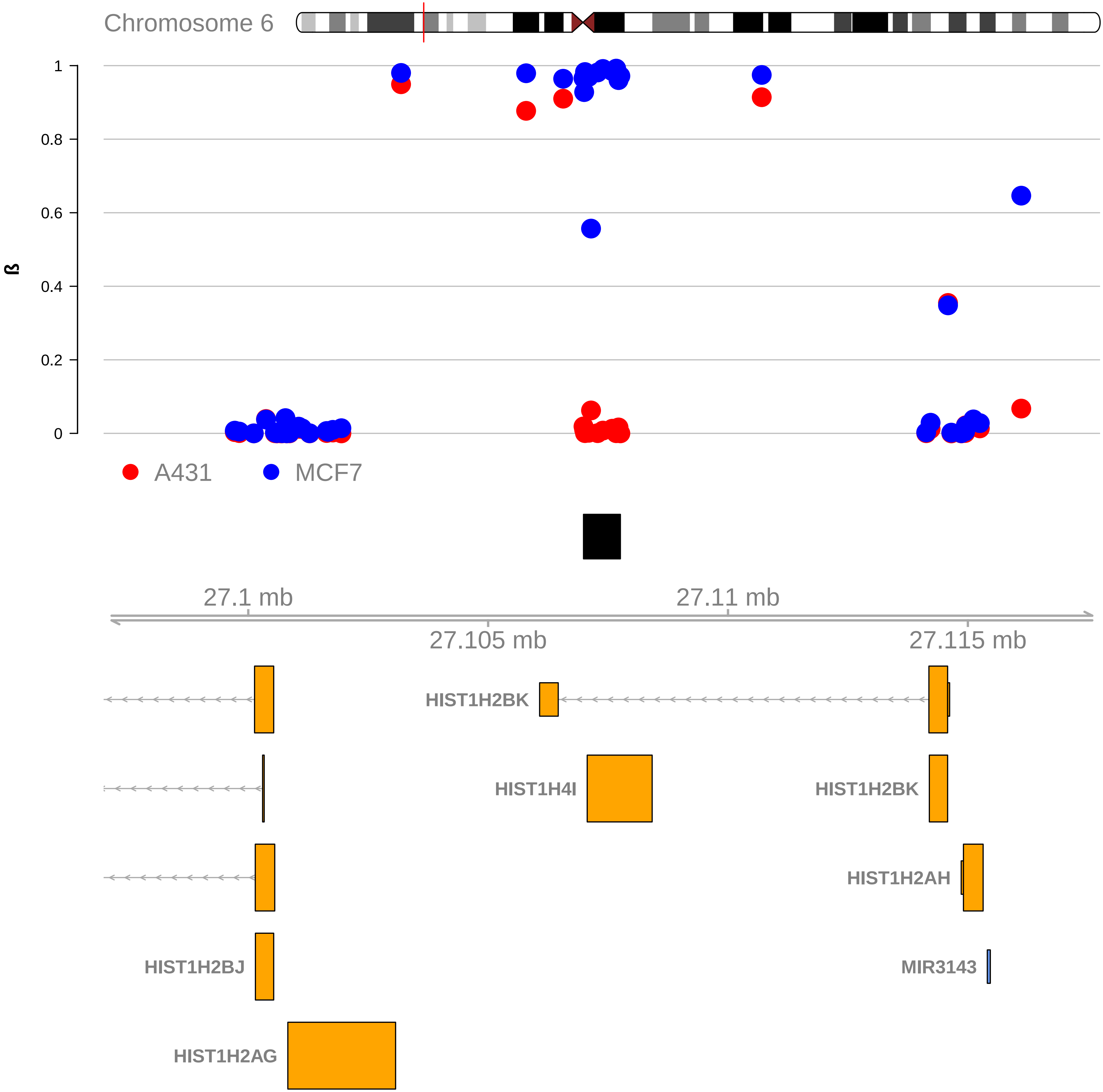

Chromosome 6
1
0.8
0.6
0.4
0.2
0
ß
A431
MCF7
27.1 mb
27.11 mb
27.105 mb
27.115 mb
HIST1H2BK
HIST1H4I
HIST1H2BK
HIST1H2AH
HIST1H2BJ
MIR3143
HIST1H2AG
